# Impact of pre-event testing and quarantine on reducing the risk of COVID-19 epidemic rebound: a modelling study

**DOI:** 10.1186/s12879-021-06963-2

**Published:** 2022-01-24

**Authors:** Ngai Sze Wong, Shui Shan Lee, Kate M. Mitchell, Eng-kiong Yeoh, Cheng Wang

**Affiliations:** 1 Stanley Ho Centre for Emerging Infectious Diseases, The Chinese University of Hong Kong, Shatin, Hong Kong, China; 2 MRC Centre for Global Infectious Disease Analysis, Department of Infectious Disease Epidemiology, Imperial College London, London, UK; 3 Centre for Health Systems and Policy Research, The Chinese University of Hong Kong, Shatin, Hong Kong, China; 4 Jockey Club School of Public Health and Primary Care, The Chinese University of Hong Kong, Shatin, Hong Kong, China; 5 Dermatology Hospital of Southern Medical University, No. 2 Lujing Road, Guangzhou, 510095 Guangdong China; 6 Southern Medical University Institute for Global Health and Sexually Transmitted Diseases, Guangzhou, Guangdong China

**Keywords:** COVID-19, Large-scale event, Testing, Quarantine, Mathematical modelling

## Abstract

**Background:**

With the evolving growth of the COVID-19 epidemic, travel restriction policies would need to be adjusted accordingly. Prohibition of mass event may be relaxed for social and economic benefits when virus transmission stops but could bear the risk of epidemic rebound. Against the background of the varied SARS-CoV-2 prevalence internationally, we modelled the potential impacts of pre-event interventions on epidemic risk of holding a mass event when COVID-19 is under control.

**Methods:**

We developed a mathematical model of SARS-CoV-2 transmission in Guangdong Province, China, where local virus transmission ceased to occur. A large-scale international trade fair was assumed to be held, with influx of people from overseas and rest of China over a short period of time, who participated for 2-week. Scenarios of pre-event intervention (none, quarantine arrangement and polymerase chain reaction (PCR) testing for participants) were compared. The influence of contact pattern, SARS-CoV-2 prevalence outside the province and China, and testing coverage were examined in sensitivity analyses.

**Results:**

In basecase scenario (no event), the epidemic has been under control since March 2020. The event would lead to the detection of 1% more confirmed cases by 31 July when community contact rate increases to pre-epidemic level. In event scenario without additional interventions, there would be 599 (93%) more new infections comparing with basecase scenario. To avert new infections, quarantining all participants before the event would be the most effective strategy, followed by quarantining all overseas participants and testing all other participants, and testing all participants before the event and on day 7. However, testing strategy is likely to be affected by the SARS-CoV-2 prevalence outside the event province.

**Conclusions:**

Pre-event interventions are effective for reducing the risk of epidemic rebound caused by an international large-scale event. Universal testing for participants is likely to be an effective and feasible intervention.

**Supplementary Information:**

The online version contains supplementary material available at 10.1186/s12879-021-06963-2.

## Background

The epidemic of COVID-19 varied temporally and geographically. In December 2020, most places in the Western Pacific were classified as belonging to the level of ‘cluster of cases’ while other places such as North America and Europe were classified as ‘community transmission’ by the World Health Organization [1]. International travel restriction have been adopted in most places to suppress the number of imported cases. A wide range of non-pharmaceutical interventions have also been implemented in different degrees over time to control the epidemic. One of the interventions is restriction of mass gathering and mass events, the setting of which involved a large number of people who may not be known to each other and are in contact for extended duration in indoor environment [2]. Outbreaks have been reported in mass events in the earlier phase of the COVID-19 pandemic, as exemplified by clusters in a business conference in Singapore [3], and a Muslim missionary movement in Malaysia [4]. In coping with the pandemic, major international activities, such as large-scale trade, politics, religious, cultural, academic and sports events, have been cancelled, postponed or become virtual worldwide [5].

When the COVID-19 epidemic was under control locally, intervention strategies were often adjusted to reduce the negative impacts in social and economic aspects [6, 7]. For instance, efforts have been made to partially relax the restriction by implementing travel bubbles for people between selected places for enabling re-connections [8]. Local gathering restrictions have been lifted, enabling organization of local mass events. However, international large-scale events are generally disallowed in consideration of the anticipated risk for widespread transmission.

To inform COVID-19 epidemic control strategies, mathematical modelling is a useful approach for risk assessment and simulation of possible outcomes under different intervention scenarios. Previous modelling studies have analyzed the impact of non-pharmaceutical interventions including contact tracing, testing, and quarantine. Interventions for special settings such as refugee camp have also been analyzed in models [9]. Exit strategies for COVID-19 were examined in a modelling study in Singapore [10]. So far, investigations specifically for mass event setting have not yet been fully explored in modelling studies. Against this background, we undertook to analyze the potential epidemiologic impacts of organizing a large-scale international event under different intervention strategies. The hypothetical scenarios of holding the China Import and Export Fair (Canton Fair) in Guangdong Province of China, a trade event attracting more than 180,000 participants from more than 40 countries per year, were developed.

## Methods

### Overview

We developed a basecase deterministic compartmental model to simulate COVID-19 epidemic under implementation of interventions and gathering restrictions in Guangdong Province, China from 28 December 2019 to 31 August 2020. We then developed hypothetical scenarios of holding a large-scale international event (event hereafter) involving participants from Guangdong, other provinces in Mainland China, and overseas, and other Guangdong residents not participating the event. Sensitivity analyses were performed to analyze the epidemic impacts of key model parameters.

### Study area

Guangdong, a province with 113.46 million population in 2018 [11], has reported 1641 confirmed cases including 8 deaths by 30 June 2020 [12]. Imported cases, defined as COVID-19 patients whose infection originated outside Guangdong, accounted for around 76% of confirmed cases [12]. Contact tracing, testing, confirmed case isolation, and quarantine were implemented throughout the epidemic period. COVID-19 epidemic in Guangdong has been under control with less than 2 daily new confirmed cases since 21 March 2020.

### Basecase model structure and assumptions

**Fig. 1:**
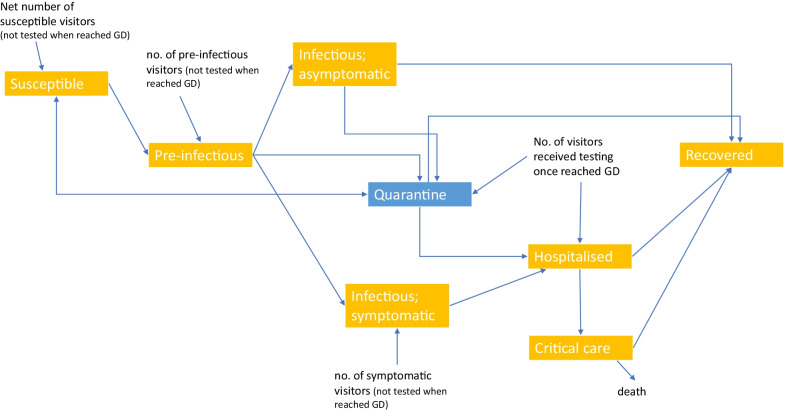
Basecase model diagram

This is an open model with individuals entering and leaving the province (Fig. 1; Additional file 1: Appendix p. 2). Besides quarantining close contacts, health quarantine of inbound travelers from listed origins have been imposed upon travelers’ arrival, and polymerase chain reaction (PCR) testing for SARS-CoV-2 (95% sensitivity) performed. We assumed all symptomatic travelers testing positive were directed to the hospital compartment, while those testing negative or positive without symptoms were directed to the quarantine compartment. After 14-days’ quarantine, individuals diagnosed would be directed to the hospital compartment, while the rest flowed back to the susceptible compartment. The quarantine arrangement applied to travelers who were from (a) Hubei Province, China, between 14 February 2020 and 23 March 2020; (b) overseas since 1 March 2020; and (c) Hong Kong and Macau, both Special Administrative Regions (SAR) of China, as from 27 March 2020. A net proportion of travelers, who were susceptible and not fulfilling quarantine criteria at the time of arrival, entered the susceptible compartment. Infected individuals not fulfilling quarantine criteria at the time of arrival (i.e. not in the list of designated countries) entered either the pre-infectious compartment or symptomatic compartment.

### Data source

Model inputs included reproduction number (R_0_), biological parameters (latent period, time to recovery in asymptomatic infections, time from hospitalization and intensive care units (ICU) to recovery in symptomatic infections, mortality rate of COVID-19, asymptomatic proportion), demographic parameters (permanent resident population in 2018 in Guangdong, travelers staying overnight in 2018 in Guangdong), policy and healthcare system parameters (contact tracing, quarantine, and testing), and event parameters (Additional file 1: Table S1). Other interventions such as school closure, group gathering restrictions, and business and premises restrictions are absorbed in background and reflected by the proportion of contact rate reduction comparing with pre-epidemic level. Model parameter values or ranges were derived from provincial yearbooks, local government reports, scientific literature and assumptions [13–18]. We fitted model predictions over time to daily number of confirmed cases in the province by 29 March 2020, using mle function under negative log likelihood in R (stats4 package). In this calibration process, we simultaneously varied the proportion of asymptomatic infection and proportion of reduction of contact rate comparing with pre-epidemic level. Modelling results were validated with the daily number of confirmed cases between 30 March and 15 May 2020. IRB approval and a waiver of consent were obtained from Dermatology Hospital of Southern Medical University, China.

### Event scenarios

The setting of the event was the biannual Canton Fair, which was organized virtually in 2020 because of the COVID-19 epidemic. Event scenario was developed on the assumption that this Fair was held on-site in a large conference venue in Guangzhou between 1 and 14 June 2020. In the event, we assumed the contact rate among participants doubled with reference to that in the community. Assuming only half of the overseas participants (100,000) in the past two years have joined the event, and the number of local participants remained the same as in the past for Guangdong (38,000) and other provinces of China (100,000), six scenarios were developed to examine the impacts of different pre-event interventions of quarantine and testing (assumptions in Additional file 1: Appendix p .2).

1. None of participants were quarantined before the event, unless they were contact traced;
2. All participants travelling from overseas were quarantined for 14 days before the event;
3. All participants (regardless of origins) were quarantined before the event;
4. Scenario 2, and all Mainland participants were tested before attending the event;
5. Scenario 1, and all participants were tested before attending the event;
6. Scenario 1, and all participants were tested before and on day 7 following opening of the event.

### Model outcomes

The main modelling outcomes were the cumulative number and proportion change of cumulative new infections generated locally (regardless of symptom presentation) above basecase scenario by 31 July 2020, to allow enough time for demonstrating the impact on incidence caused by the event. The secondary outcome was the cumulative number of confirmed cases.

### Sensitivity analyses

To account for variability of R_0_, contact rate, and proportion of asymptomatic infections over time, we have performed 5000 simulations in the basecase model (Additional file 1: Appendix p .18). We performed one-way sensitivity analysis around the key parameters in the following scenarios: contact rate reduction from baseline ranged between 0 and 90% since 1 May 2020, prevalence of SARS-CoV-2 in overseas participants (0.00005–0.001) and other provinces’ participants (0.00001–0.005), proportion of all infected individuals in Guangdong who attended the event (0.001–0.1), and testing coverage (0–100%) for participants in scenarios 4–6. We also varied the contact rate at the event from doubled, tripled and ten-fold with reference to that in the community. To examine the impact of duration with very low contact rate (10%) through intense interventions at epidemic peak (9 February 2020) and the change of contact rate after relaxing the interventions, two-way sensitivity analysis was performed.

## Results

### Basecase scenario

**Fig. 2:**
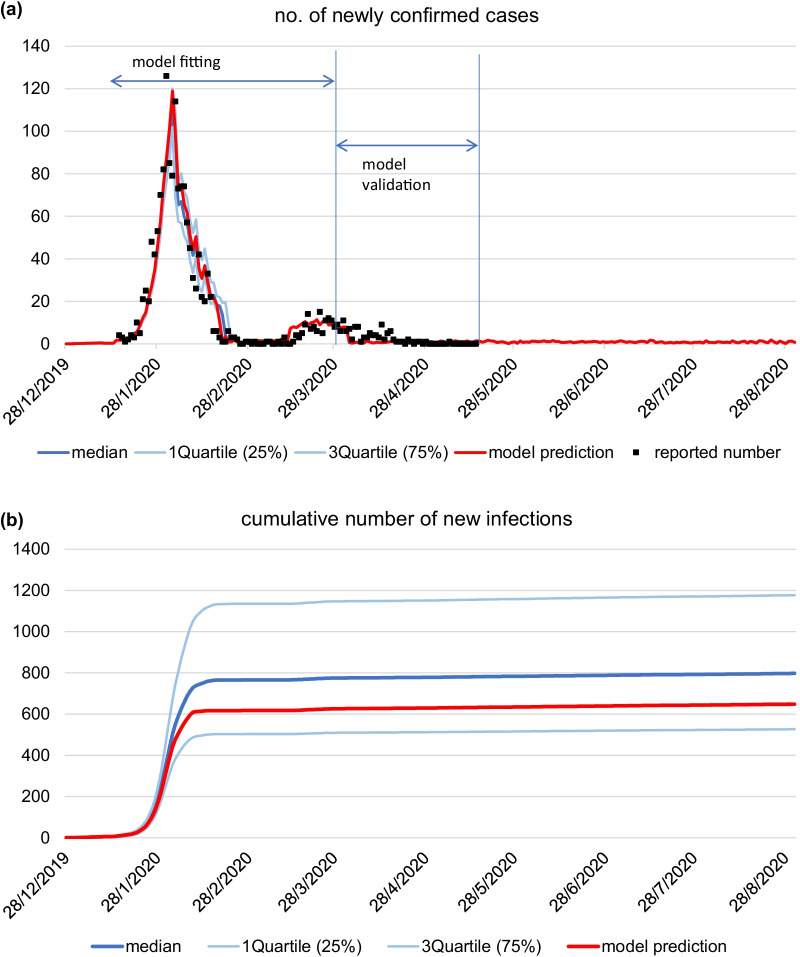
Model estimates of (**a**) daily number of new COVID-19 confirmed cases and (**b**) cumulative number of new SARS-CoV-2 infections in Guangdong Province, 28 December 2019–31 August 2020

Our basecase model (without event) provided a reasonable fit to the daily number of newly confirmed (symptomatic only) cases in Guangdong from 15 January to 29 March 2020, and prediction from 30 March to 15 May 2020 (Fig. 2). In basecase scenario, the daily number of confirmed cases reached its peak in early February and declined rapidly within two weeks. The model estimated that the number of cumulative local new infections reached the plateau of between 600 and 650 in mid-February. By 31 July, the estimated cumulative number of new infections and confirmed cases would be 644 and 1680, respectively.

**Fig. 3:**
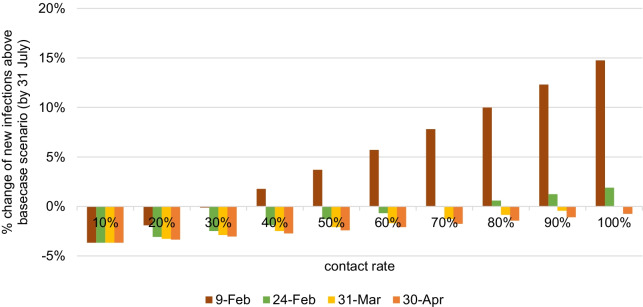
Impact of contact rate and time of changing the contact rate in two-way sensitivity analysis Percentage change of cumulative number of new infections above basecase scenario by 31 July 2020, along the change of contact rate (10–100% comparing with pre-epidemic level in x-axis) from dates of 9 February, 24 February, 31 March and 30 April 2020 (shown in color scale). Parameter values used in basecase scenario is changing to 70% contact rate from 24 February 2020

To account for the changes of contact rate from pre-epidemic level, one-way sensitivity analysis was performed. By changing the contact rate from 70% (status quo) to 100% from 1 May 2020, the cumulative number of new infections and confirmed cases was 1% and 0.4% higher respectively than basecase scenario (Additional file 1: Fig. S1). On the contrary, if contact rate decreased to 10%, the estimated cumulative number of new infections and confirmed cases would be 2% and 1% lower than basecase scenario respectively. Assuming that intense intervention had not achieved very low contact rate (10% of pre-epidemic level) from 9 February 2020, the estimated cumulative number of new infections would be 8% higher than basecase scenario (Fig. 3; Additional file 1: Fig. S2), as shown in two-way sensitivity analysis.

### Event scenario

**Fig. 4:**
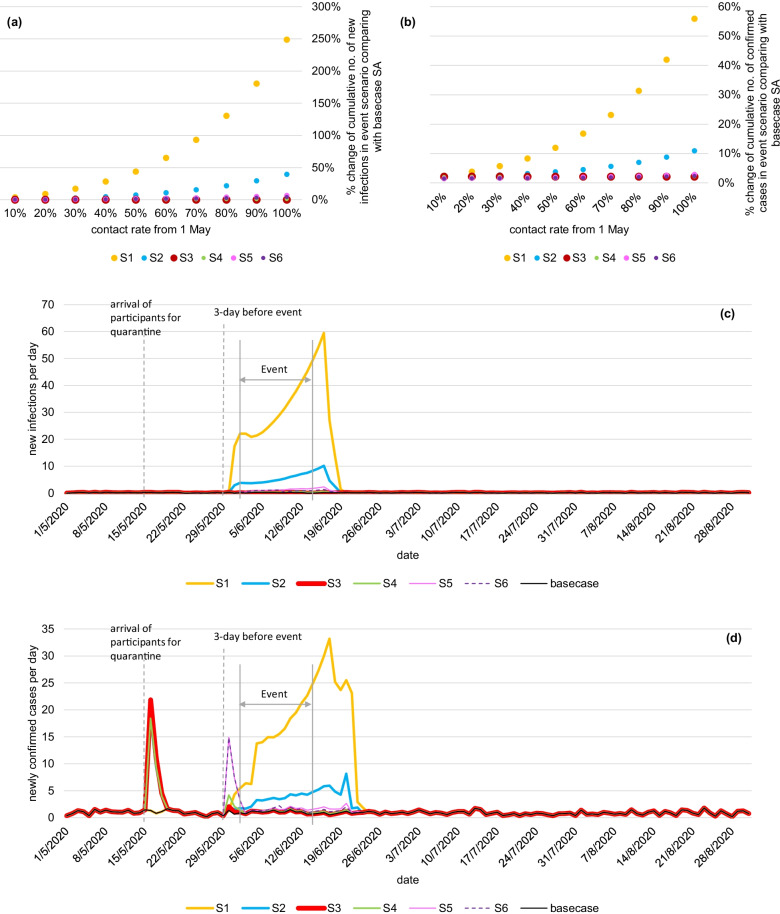
Impact of Canton Fair on epidemic with different interventions under different contact rate reduction background. the percentage change of (**a**) cumulative number of new infections and (**b**) cumulative number of confirmed cases by 31 July 2020 under different contact rate from 1 May 2020 in different event scenario; and the daily number of (**c**) new infections and (**d**) newly confirmed cases in different event scenarios with contact rate remained to be 70% of pre-epidemic level

If the Canton Fair was organized without any pre-event interventions and the contact rate remained at 70% of pre-epidemic level (scenario 1), the estimated cumulative number of new infections and confirmed cases would be 93% higher (599 more infections) and 23% higher (388 more cases) than basecase scenario by 31 July 2020, respectively (Fig. 4a, b). The daily number of new infections and confirmed cases would increase linearly in the event period, reaching the peak after three days, following which local participants would have spent all time in the community (Fig. 4c, d). If all overseas participants were quarantined before the event (scenario 2), the estimated cumulative number of new infections would be 16% higher than basecase scenario. By expanding the quarantine criteria to all participants (scenario 3), no new infections attributable to the event was estimated. However, the total number of confirmed cases would be 2% higher, all of which confirmed once they arrived at the province/event venue (Fig. 4b, d). When all overseas participants were quarantined while all participants from Mainland China were tested before the event (scenario 4), the estimated total new infections would be 1% higher than basecase scenario. Comparing with basecase scenario, the estimated number would be 3% higher if all participants were tested before the event (scenario 5), and 2% higher if all participants were tested both before the event and on day 7 of the event. In scenarios involving testing strategies, a decrease of the testing coverage from 100 to 10%, would lead to 14%, 81%, and 79% of additional infections in scenarios 4, 5, and 6, respectively (Additional file 1: Fig. S3).

Sensitivity analysis was performed on various scenarios when contact rate was back to the pre-epidemic level (i.e. 100%) from 1 May 2020. Compared to basecase scenario, organizing the event would result in no change in the estimated cumulative number of new infections under scenario 3, minimal change (1% higher than basecase scenario) in scenario 4, and less than 10% more new infections under scenarios 5–6 (Fig. 4). However, the cumulative number of new infections would be 39% higher than basecase scenario in scenario 2 by quarantining overseas participants only, and 2.5 folds higher without interventions (scenario 1). The model has assumed that participants would double their contacts in the event comparing with that in the community. When the contact rate at the event is triple or ten-fold higher, substantial increases in total new infections were estimated in all scenarios except scenario 3. In scenario 1 without pre-event interventions, the estimated total number of new infections by 31 July 2020 would be 235% and 13,339% higher than basecase scenario if contact rate in the event was triple and ten-fold higher, respectively (Additional file 1: Table S2).

The impact of the SARS-CoV-2 prevalence in other provinces and overseas on the percentage change of total new infections would be high, as shown in sensitivity analyses. The cumulative number of new infections in scenario 1 would rise from 80% higher to 350% higher than basecase scenario when the prevalence in other provinces increased from 0.00001 to 0.001 (Additional file 1: Fig. S4). When prevalence in other provinces rose from 0.00001 to 0.001 in scenario 2, 264% more new infections were estimated. Similarly, there would be 253% more new infections in scenario 1 than basecase scenario if the prevalence overseas increased from 0.00005 to 0.001. However, there would be minimal impact (< 2% change on total new infections) when proportion of local infected cases participating in the event changed from 0.1% to 10% in all scenarios.

## Discussion

Since the emergence of the COVID-19 pandemic, intense interventions have been implemented to minimize gathering of people and travel between places [10, 19, 20]. Large-scale international events were cancelled or postponed, resulting in epidemic control but anticipated economic loss. This modelling study simulated the potential epidemiologic impact of organizing an international trade event with and without pre-event interventions. At basecase without event, the epidemic is under control even if the contact rate increased to the pre-epidemic level in Guangdong, China. Without additional pre-event interventions, organizing the Canton Fair at the end of the local epidemic could double the cumulative number of new infections if SARS-CoV-2 prevalence in other provinces is 0.00006 and overseas is 0.0003. The increase of new infections would be proportional to the prevalence during event organization.

Our study suggested that additional pre-event interventions including quarantine and testing for participants could avert a substantial proportion of new infections. Quarantining all participants regardless of origins would be most effective. It is least likely to be affected by other factors including community contact rate and SARS-CoV-2 prevalence in other places. Such positive outlook is achievable if all infected cases could be identified, quarantined or hospitalized before the event. There would be minimal chance for outbreaks in the community and at the event venue. The effectiveness of quarantine intervention on epidemic growth is consistent with the results of previous modelling studies [15, 21, 22]. However its feasibility is doubtful because of capacity need to quarantine more than 0.2 million people for 14 days at the same time.

Testing all Mainland participants and quarantining all overseas participants (scenario 4) would be similarly effective as the strategy of quarantining all participants (scenario 3), if the prevalence in other provinces remains low. The strategy of testing everyone (scenario 5) would result in slightly higher proportion of total new infections (~ 2%) but could keep the quarantined number at the minimum. However, the impact of testing intervention would be affected by the current state of the epidemic, and the capability of detecting pre-infectious cases through testing. At higher prevalence, the estimated number of new infections would be larger even the rise would not be as high as of scenario 2 (quarantining overseas participants but no intervention for Mainland participants). Strategically adding one more time point for testing all participants (scenario 6) could avert a proportion of new infections estimated in scenario 5. The effectiveness of testing intervention to avert new infections was shown in a modelling study [23], suggesting that universal testing alone without lockdown could reduce the amplitude of the peak by 40% [23]. Universal testing has been suggested as a lockdown exit strategy in the United Kingdom, with feasibility study planned [24]. With improvement of sensitivity and specificity of SARS-CoV-2 tests and availability of point-of-care tests [24], accurate and timely test results would be available to facilitate efficient implementation of control. This would increase the feasibility of positioning universal testing as a key preventive intervention to minimize adverse economic impacts of COVID-19.

The infection risk of an international event rests not just with potential virus exposure at the event setting but also transmission in the community. With low contact rate in the community, limited ongoing transmissions could occur in association with exposure to imported cases. The difference of estimated new infections with and without different pre-event interventions would be small. However, when community contact rate before the event approaches the pre-epidemic level, the likelihood of ongoing transmission could be higher. To control possible ongoing transmissions from imported cases, pre-event intervention strategies would play a key role. The importance of contact rate on epidemic growth could be reflected from the implementation of physical distancing measures in the real-world with effectiveness shown in modelling studies [25–27]. To minimize the adverse impact of organizing an event, the contact rate in the community would have to be taken into consideration.

There are a few limitations in this modelling study. First, the logistics for local quarantine arrangement, and the provision of SARS-CoV-2 tests (on days 1, 4, 7 and 14) have been simplified [13]. Nonetheless, with limited number of quarantined persons eventually diagnosed with COVID-19, the impact of simplified logistics could be minimal. We also acknowledged the possibility of introducing new infections in household members of home-quarantined individuals for residents in the organizer city, the occurrence of which has been absorbed in the background. Second, we used contact rate in comparison with pre-epidemic level to evaluate the overall impacts of physical distancing measures in the community. There were no further breakdowns of contact rate reduction as differentiated by the types of measures, such as school closure, workplace closure, and restriction of gathering in public area, while only quarantine and PCR testing for SARS-CoV-2 were specified as pre-event interventions in modelling scenarios. We acknowledged the difficulties and inherent limitations of evaluating the specific impacts of different contact rates and their association with respective social distancing measure. Third, the event scenarios had assumed that many overseas individuals would still participate in the event despite the 14-day pre-event quarantine. Although we assumed a 50% reduction of the number of overseas participants comparing with the previous years, the actual number may be even lower, and the model may have overestimated the number of new infections in the event scenarios. We have also oversimplified the flow of participants coming in and leaving the event by assuming that all participants would stay for the whole period. The downside, again, was the overestimation of the epidemic impacts of the model. Finally, the modelling study has focused on the growth of the COVID-19 epidemic at an international event that took place after the outbreak has gone to a quiescent phase. Other public health impacts, for example, cost-effectiveness analysis may be needed to account for the cost and utility involved in each strategy.

## Conclusion

When restriction of international large-scale event organization is lifted, different forms of pre-event interventions could be considered to effectively reduce the risk of widespread SARS-CoV-2 transmission. Mathematical modelling is a useful approach for risk assessment and planning of pre-event interventions.

## Supplementary Information

**Additional file 1**: **Appendix**.

## Data Availability

The datasets used and/or analysed during the current study are available from the corresponding author on reasonable request.

## Abbreviations

ICU: Intensive care units
PCR: Polymerase chain reaction
R_0_: Reproduction number
