# Supplementary material for: Impact of pre-event testing and quarantine on reducing the risk of COVID-19 epidemic rebound: a modelling study
Source: BMC Infect Dis. 2022 Jan 24;22:83. doi: 10.1186/s12879-021-06963-2 (PMC8785033; doi:10.1186/s12879-021-06963-2)
Supplement: Supplementary file 1 — Additional file 1: Appendix. [file 12879_2021_6963_MOESM1_ESM.docx]

**Impact of pre-event testing and quarantine on reducing the risk of COVID-19 epidemic rebound: a modelling study**

**Ngai Sze Wong, Shui Shan Lee, Kate M. Mitchell, Eng-kiong Yeoh, Cheng Wang**

**Appendix**

**[Model description](#_Toc59095890)** [2](#_Toc59095890)

**[eTable 1 List of model parameters](#_Toc59095891)** [4](#_Toc59095891)

**[Equations for basecase model](#_Toc59095892)** [7](#_Toc59095892)

**[Equations for event scenarios](#_Toc59095893)** [10](#_Toc59095893)

**[Maximum likelihood estimation](#_Toc59095894)** [19](#_Toc59095894)

**[Sensitivity analysis](#_Toc59095895)** [19](#_Toc59095895)

**[eTable 2 Sensitivity analysis: contacts tripled and decupled in the event comparing with the community](#_Toc59095896)** [20](#_Toc59095896)

**[eFigure 1 Sensitivity analysis: impact of contact rate](#_Toc59095897)** [21](#_Toc59095897)

**[eFigure 2 Two-way sensitivity analysis: date of changing to higher contact rate and level of contact rate](#_Toc59095898)** [22](#_Toc59095898)

**[eFigure 3 Sensitivity analysis: impact of testing coverage in scenarios 4-6](#_Toc59095899)** [23](#_Toc59095899)

**[eFigure 4 Sensitivity analysis: impact of SARS-CoV-2 prevalence in participants before the event](#_Toc59095900)** [24](#_Toc59095900)

**[Reference](#_Toc59095901)** [25](#_Toc59095901)

# **Model description**

**Basecase model structure and assumptions**: In basecase model (no event), susceptible individuals became infected at a rate dependent on reproduction number (R_0_), duration of infectiousness for asymptomatic and symptomatic infections, and proportion of population who were infectious (parameter value in **eTable 1**). The general impact of physical distancing measures was represented by the reduction of contact rate comparing with pre-epidemic level. Following the latent period (also known as pre-infectious period), infected individuals transited from pre-infectious to either asymptomatic (infectious) or symptomatic (infectious) compartment (**Figure 1**). With contact tracing, susceptible, pre-infectious and asymptomatic individuals were quarantined for 14-days. Asymptomatic individuals (with or without quarantine) were assumed to recover without hospitalization, while symptomatic individuals were diagnosed and transited to the hospital for treatment and isolation. Some hospitalized individuals with severe condition were transferred to intensive care units (ICU) while the rest recovered. The model excluded nosocomial infection as no transmission in the healthcare settings had been reported in the province.

**Event assumptions**: In the model, staff and attenders were grouped as “participants”. The force of SARS-CoV-2 infection was assumed to be different at the event and in the community. Participants would spend an estimated eight hours (two-thirds of time outside living place) at the event and four hours (one-third of time) in the community per day. Contacts among participants at the event were assumed to be double that in the community. In scenarios with quarantined participants before event, participants were assumed to arrive 15-17 days before the opening, while those not requiring quarantine arrived 1-3 days in advance. In scenarios 4-6, PCR testing was performed at the airport/control points for overseas participants and other Mainland provinces’ participants upon arrival, and at the event venue for Guangdong participants. Asymptomatic individuals tested positive would have been quarantined while symptomatic individuals tested positive would be treated in hospitals with isolation. All participants were assumed to attend the whole period of the Fair unless otherwise specified. Participants from other provinces and overseas were assumed to leave the event venue within three days. Overseas participants were not further categorized by their country of origin because of insufficient data to differentiate their contact rates. The SARS-CoV-2 prevalence was assumed to be similar to that in mid-April 2020 (i.e. 0.00006 in other provinces and 0.0003 in overseas).

# **eTable 1 List of model parameters**

| **Description** | **Symbol** | **Estimated value/fitted value** | **Remarks (Source)** |
| --- | --- | --- | --- |
| **Biological parameters** | | | |
| Reproduction number (average number of secondary cases that each case generates)[3] | R0 | 2.2 | [1] |
| Latent period (from infected to pre-infectious)[3] | latent | 4.7 days | [2] |
| Duration of infectious before symptom onset | Infb4onset | 0.5 days | [3] |
| Proportion of asymptomatic infection | asyp | 0.34 [fitted] | Varied from 0.2 to 0.8 for model calibration |
| Rate of recovery after hospitalization | 1/r | 1/8 days | [3] |
| Proportion of hospitalized individuals being transferred to ICU | cc | 0.13 (0.1-0.16) | Local government report |
| Rate of recovery for asymptomatic infection | 1/r2 | 1/6 days | Local government report |
| Mortality rate for COVID 2019 (ICU patients) | m | 8/1484 | Surveillance data |
| **Demographics** | | | |
| Guangdong permanent population in 2018 | GDpop | 98 555 550 | Guangdong Province Yearbook 2019 |
| Number of travelers staying overnight in Guangdong Province in 2018 | S_travelers | 37 480 592 person /365 days from overseas **+** 452 527 902 person /365 days from other provinces in China | Guangdong Province Yearbook 2019  We assumed the proportion of these overseas and other provinces’ travelers entering the model varied over time after the first reported cases (reduced by 20% in 11 January-12 February, reduced by 95% from 12 to 29 February, and only limited to other mainland places and reduced by 90% from 1 March 2020 |
| Net proportion of travelers staying in Guangdong Province | SS | 0.01 | Assumption |
| Number of infected travelers entering Guangdong Province without testing upon arrival | vrHB |  | Represented by number of imported cases with 3 days’ lag before 1 March 2020, and multiplied by 0.3 afterwards as 70% of these imported cases are assumed to receive testing upon arrival from 1 March |
| **Policy / healthcare system parameters** | | | |
| Delay of diagnosis after symptom onset | dx | 3.2 days | [4] |
| Quarantine duration | flow | 14 days |  |
| number of confirmed cases after quarantine / total number of quarantined cases | Qdx | 0.00000000000001 | Assumption |
| Number of individuals identified through contact tracing | flowtracing | Daily number of confirmed cases x closec |  |
| Number of testing for travelers once arrived the province | Qtesting | 14-17 Feb: 8846  18-23 Feb: 12715  24 Feb-11 Mar: 786  12-31 Mar: 2260  1 Apr onwards: 100 | Local government report |
| Proportion of travelers tested positive | posqtest | 14-17 Feb: 0.00026  18-29 Feb: 0.000069  1-11 Mar: 0.0018  12-31 Mar: 0.0031  1 Apr onwards: 0.00155 | Local government report |
| Average number of close contacts per index case | closec | 5 | [4] |
| Proportion of close contacts tested positive | closepos | 0.068 | [4] |
| Proportion of cases developed symptom after quarantine | closepreinf | 0.05 | [4] |
| Proportion of close contacts tested positive who were symptomatic | closepos_sym | 0.8 | [4] |
| Estimated effective reduction of contact rate by different control policies, comparing with before epidemic | controleff | Under the first level emergency response,[5] contact rate was reduced by 90%/3 comparing with baseline between 22 January and 8 February 2020, and reduced by 90% between 9 and 23 Feb; under the second level emergency response,[6] contact rate was reduced by 90%/3=30% from 24 Feb 2020 onwards [fitted] | Varied from 0 to 0.9 for model calibration |
| Proportion of imported cases entering the province being symptomatic | symHB | 0.64 | [4] |
| **Event scenario parameter** | | | |
| number of participants and staff from Guangdong Province | GDpar | 38000 | Average annual number of overseas participants in the past 2 years |
| proportion of infected individuals in Guangdong Province participating the event | GDinf | 0.01 | Assumption |
| number of participants and staff from overseas | overseaspar | 100000 | Average annual number of overseas participants in the past 2 years, and the number in scenario was assumed to be smaller due to the epidemic |
| prevalence of SARS-CoV-2 in participants and staff from overseas | seainf | 0.0003 | Estimated prevalence overseas by 19 April 2020 |
| number of participants and staff from other provinces | oPpar | 100000 | Average annual number of overseas participants in the past 2 years |
| prevalence of SARS-CoV-2 in participants and staff from other provinces | oPinf | 0.00006 | Estimated prevalence in other provinces by 19 April 2020 |
| proportion of infected participants and staff from Mainland China who are tested positive | Opos | 0.95 | 1- closepreinf |

# **Equations for basecase model**

**Force of infection**$\boldsymbol{\lambda}$

$\lambda=((1-controleff[t]) * (\frac{R0}{r2} * Ainf[t] + \frac{R0}{dx+Infb4onset} * inf[t]))/N[t]$;

*controleff* = estimated effective reduction of contact rate by different control policies, comparing with before epidemic; *dx* = delay of diagnosis after symptom onset; *R0*=reproduction number; 1/*r2* = rate of recovery for asymptomatic infection; *N* = total population; *Infb4onset* = duration of infectious before symptom onset

**Susceptible (S)**

$\frac{dS}{\mathrm{dt}}=S_{\mathrm{travelers}\left[ t \right]}*SS-\lambda*S\left[ t \right]-\mathrm{flowtracing}\left[ t \right]*\left( 1-closepos \right)+flowtracing\left[ t-14 \right]*(1-closepos)+\left( 1-Qdx \right)*Qtesting[t-14] * (1-posqtest[t-14])$;

*closepos* = proportion of close contacts tested positive; *closec* = average number of close contacts per index case; *flowtracing* = number of individuals identified through contact tracing = confirm[t-1]*closec; *postqtest* = proportion of travelers tested positive; *Qdx* = number of confirmed cases after quarantine / total number of quarantined cases; *Qtesting*= number of testing for travelers once arrived the province; *S_*travelers = number of travelers from overseas staying overnight in Guangdong Province in 2018; SS = net proportion of travelers staying in Guangdong Province

**Pre-infectious (preinf)**

$$\frac{dpreinf}{\mathrm{dt}}=\left( 1-symHB \right)* vrHB\left[ t \right]+ \lambda*S\left[ t \right]- \left( \frac{1}{\mathrm{latent}} \right)* preinf\left[ t \right]-flowtracing\left[ t \right]*closepos*closepos\_sym*closepreinf;$$

closepreinf = proportion of cases developed symptom after quarantine*; latent* = latent period; *symHB*= proportion of imported cases entering the province being symptomatic; *vrHB* = number of infected travelers entering Guangdong Province without testing upon arrival;

**Asymptomatic infection (Ainf)**

$$\frac{dAinf}{\mathrm{dt}}=asyp * \left( \frac{1}{\mathrm{latent}}* preinf\left[ t \right] \right)- \frac{1}{r2}* Ainf\left[ t \right]-flowtracing\left[ t \right]*closepos*(1-closepos\_sym);$$

*asyp* = proportion of asymptomatic infection; *closepos_sym* = proportion of close contacts tested positive and were symptomatic

**Symptomatic infection (inf)**

$$\frac{dinf}{\mathrm{dt}}=symHB* vrHB[t] + (1-asyp) * (\frac{1}{\mathrm{latent}} * preinf[t]) -\frac{1}{dx+Infb4onset}* inf[t]-flowtracing\left[ t \right]*closepos*closepos\_sym*(1-closepreinf);$$

**Quarantine (QGD)**

$$\frac{dQGD}{\mathrm{dt}}=\mathrm{flowtracing}\left[ t \right]*\left( 1-closepos \right)+flowtracing\left[ t \right]*closepos*\left( 1-\mathrm{closepos}_{\mathrm{sym}} \right)+\mathrm{flowtracing}\left[ t \right]*closepos*\mathrm{closepos}_{\mathrm{sym}}*closepreinf+ Qtesting\left[ t \right]* \left( 1-posqtest\left[ t \right] \right)-\left[ \mathrm{flowtracing}\left[ t-14 \right]*\left( 1-closepos \right)+ Qtesting\left[ t-14 \right]* \left( 1-posqtest\left[ t-14 \right] \right)+flowtracing\left[ t \right]*closepos*\left( 1-\mathrm{closepos}_{\mathrm{sym}} \right)+flowtracing\left[ t-3 \right]*closepos*\mathrm{closepos}_{\mathrm{sym}}*closepreinf \right];$$

all quarantined individuals were assumed to complete the whole quarantine duration (14 days), except for some contact traced individuals developed symptoms after quarantine (assuming 3 days)

**Hospital (Hosp)**

$$\frac{dHosp}{\mathrm{dt}}=\frac{1}{dx+Infb4onset}* \left( \inf\left[ t \right] \right) -\left( cc+\frac{1}{r} \right)* Hosp\left[ t \right]+ \left( 1-asyp \right)* Qdx *Qtesting\left[ t-14 \right]* \left( 1-posqtest\left[ t-14 \right] \right)+flowtracing\left[ t \right]*closepos*closepos\_sym*(1-closepreinf) + Qtesting[t] * posqtest\left[ t \right]+flowtracing\left[ t-3 \right]*closepos*closepos\_sym*closepreinf;$$

*cc*= proportion of hospitalised individuals being transferred to ICU; r = rate of recovery after hospitalisation

**ICU (ICU)**

$\frac{dICU}{\mathrm{dt}}=cc * Hosp\left[ t \right]- \left( \frac{1}{r}+ m \right)* ICU\left[ t \right];$

m = mortality rate for COVID 2019 (ICU patients) in Guangdong Province

**Recovery (Recovery)**

$\frac{\mathrm{dRecovery}}{\mathrm{dt}}=\frac{1}{r}*(Hosp\left[ t \right]+ ICU[t]) + \frac{1}{r2}* Ainf[t]+asyp* Qdx * Qtesting[t-14] * (1-posqtest[t-14]) +flowtracing\left[ t-14 \right]*closepos*(1-closepos\_sym) ;$

**Death (death)**

$\frac{\mathrm{dDeath}}{\mathrm{dt}}=m* ICU\left[ t \right];$

**Counter for number of newly confirmed cases (confirm)**

$\frac{\mathrm{dconfirm}}{\mathrm{dt}}=\frac{1}{dx+Infb4onset}* \left( \inf\left[ t \right] \right) + \left( 1-asyp \right)* Qdx *Qtesting\left[ t-14 \right]* \left( 1-posqtest\left[ t-14 \right] \right)+flowtracing\left[ t \right]*closepos*closepos\_sym*(1-closepreinf) + Qtesting[t] * posqtest\left[ t \right]+flowtracing\left[ t-3 \right]*closepos*closepos\_sym*closepreinf;$

**Counter for number of newly infected cases (GDinf)**

$$\frac{\mathrm{dGD}inf}{\mathrm{dt}}=\lambda*S\left[ t \right];$$

# **Equations for event scenarios**

**Force of infection**$\boldsymbol{\lambda}$

$\lambda=((1-controleff[t]) * (\frac{R0}{r2} *(Ainf\left[ t \right]+AinfEvent\left[ t \right]+AinfEventO\left[ t \right])+ \frac{R0}{dx+Infb4onset} *(inf\left[ t \right]+infEvent\left[ t \right]+infEventO[t])))/(N\left[ t \right]+eventN\left[ t \right])$;

*controleff* = estimated effective reduction of contact rate by different control policies, comparing with before epidemic; *dx* = delay of diagnosis after symptom onset; *R0*=reproduction number; 1/*r2* = rate of recovery for asymptomatic infection; *N* = total population; *Infb4onset* = duration of infectious before symptom onset

**Force of infection for participants**$\boldsymbol{\lambda p}$

$\lambda p=\frac{\left( 1-controleff\left[ t \right] \right) * \frac{2}{3}*2*\left( \frac{R0}{r2} *\left( \mathrm{AinfEvent}\left[ t \right]+AinfEventO\left[ t \right] \right)+ \frac{R0}{dx+Infb4onset} *\left( \mathrm{infEvent}\left[ t \right]+infEventO\left[ t \right] \right) \right)}{\mathrm{eventN}\left[ t \right]}+\frac{\left( 1-controleff\left[ t \right] \right) *\frac{1}{3}* \left( \frac{R0}{r2} *Ainf\left[ t \right]+ \frac{R0}{dx+Infb4onset} *Ainf\left[ t \right] \right)}{N\left[ t \right]}$;

eventN[t] = total number of event participants from local, other provinces and overseas

N[t] = total pop in GD and all event participants

We assume participants spend 8 hours (2/3 of time outside living place) in event venue and 4 hours (1/3 of time) in the community per day. Contacts among participants in event venue are assumed to be doubled.

**Susceptible (S)**

$\frac{dS}{\mathrm{dt}}=S_{\mathrm{travelers}\left[ t \right]}*SS-\lambda*S\left[ t \right]-\mathrm{flowtracing}\left[ t \right]*\left( 1-closepos \right)+flowtracing\left[ t-14 \right]*\left( 1-closepos \right)+\left( 1-Qdx \right)*\mathrm{Qtesting}\left[ t-14 \right]* \left( 1-posqtest\left[ t-14 \right] \right)-GDpar[t]$;

*closepos* = proportion of close contacts tested positive; *closec* = average number of close contacts per index case; *flowtracing* = number of individuals identified through contact tracing = confirm[t-1]*closec; *GDpar*=number of participants and staff from Guangdong Province; *postqtest* = proportion of travelers tested positive; *Qdx* = number of confirmed cases after quarantine / total number of quarantined cases; *Qtesting*= number of testing for travelers once arrived the province; *S_travelers* = number of travelers from overseas staying overnight in Guangdong Province in 2018; SS = net proportion of travelers staying in Guangdong Province

**Pre-infectious (preinf)**

$$\frac{dpreinf}{\mathrm{dt}}=\left( 1-symHB \right)* vrHB\left[ t \right]+ \lambda*S\left[ t \right]- \left( \frac{1}{\mathrm{latent}} \right)* preinf\left[ t \right]-flowtracing\left[ t \right]*closepos*closepos\_sym*closepreinf -GDinf[t]* preinf[t];$$

*closepreinf* = proportion of cases developed symptom after quarantine*; GDinf*=proportion of infected individuals in Guangdong Province participating the event*; latent* = latent period; *symHB*= proportion of imported cases entering the province being symptomatic; *vrHB* = number of infected travelers entering Guangdong Province without testing upon arrival;

**Asymptomatic infection (Ainf)**

$$\frac{dAinf}{\mathrm{dt}}=asyp * \left( \frac{1}{\mathrm{latent}}* preinf\left[ t \right] \right)- \frac{1}{r2}* Ainf\left[ t \right]-flowtracing\left[ t \right]*closepos*(1-closepos\_sym) -GDinf[t]* Ainf[t];$$

*asyp* = proportion of asymptomatic infection; *closepos_sym* = proportion of close contacts tested positive and were symptomatic

**Symptomatic infection (inf)**

$$\frac{dinf}{\mathrm{dt}}=symHB* vrHB[t] + (1-asyp) * (\frac{1}{\mathrm{latent}} * preinf[t]) -\frac{1}{dx+Infb4onset}* inf[t]-flowtracing\left[ t \right]*closepos*closepos\_sym*(1-closepreinf)- GDinf[t]* inf[t];$$

**Quarantine (QGD)**

$$\frac{dQGD}{\mathrm{dt}}=\mathrm{flowtracing}\left[ t \right]*\left( 1-closepos \right)+flowtracing\left[ t \right]*closepos*\left( 1-\mathrm{closepos}_{\mathrm{sym}} \right)+\mathrm{flowtracing}\left[ t \right]*closepos*\mathrm{closepos}_{\mathrm{sym}}*closepreinf+ Qtesting\left[ t \right]* \left( 1-posqtest\left[ t \right] \right)-\left[ \mathrm{flowtracing}\left[ t-14 \right]*\left( 1-closepos \right)+ Qtesting\left[ t-14 \right]* \left( 1-posqtest\left[ t-14 \right] \right)+flowtracing\left[ t \right]*closepos*\left( 1-\mathrm{closepos}_{\mathrm{sym}} \right)+flowtracing\left[ t-3 \right]*closepos*\mathrm{closepos}_{\mathrm{sym}}*closepreinf \right];$$

all quarantined individuals were assumed to complete the whole quarantine duration (14 days), except for some contact traced individuals developed symptoms after quarantine (assuming 3 days)

**Hospital (Hosp)**

$\frac{dHosp}{\mathrm{dt}}=\frac{1}{dx+Infb4onset}* \left( \inf\left[ t \right] \right) -\left( cc+\frac{1}{r} \right)* Hosp\left[ t \right]+ \left( 1-asyp \right)* Qdx *Qtesting\left[ t-14 \right]* \left( 1-posqtest\left[ t-14 \right] \right)+flowtracing\left[ t \right]*closepos*closepos\_sym*(1-closepreinf) + Qtesting[t] * posqtest\left[ t \right]+flowtracing\left[ t-3 \right]*closepos*closepos\_sym*closepreinf;$

*cc*= proportion of hospitalised individuals being transferred to ICU; r = rate of recovery after hospitalisation

**ICU (ICU)**

$\frac{dICU}{\mathrm{dt}}=cc * Hosp\left[ t \right]- \left( \frac{1}{r}+ m \right)* ICU\left[ t \right];$

m = mortality rate for COVID 2019 (ICU patients) in Guangdong Province

**Recovery (Recovery)**

$\frac{\mathrm{dRecovery}}{\mathrm{dt}}=\frac{1}{r}*(Hosp\left[ t \right]+ ICU[t]) + \frac{1}{r2}* Ainf[t]+asyp* Qdx * Qtesting[t-14] * (1-posqtest[t-14]) +flowtracing\left[ t-14 \right]*closepos*(1-closepos\_sym) ;$

**Susceptible local participants (SusEvent)**

$\frac{d\mathrm{SusEvent}}{\mathrm{dt}}=GDpar[t] -\lambda p*SusEvent\left[ t \right]$; - for scenarios 1, 2, 4-6

$\frac{d\mathrm{SusEvent}}{\mathrm{dt}}=GDpar[t-14] -\lambda p*SusEvent\left[ t \right]$; - for scenario 3

Scenario 1 - None of the participants are quarantined before the event;

Scenario 2 - Only non-Mainland participants are quarantined for 14 days before the event;

Scenario 3 - All participants (regardless of origins) are quarantined for 14 days before the event;

Scenario 4 - Only non-Mainland participants are quarantined for 14 days before the event, while all Mainland participants are tested before attending the event, and asymptomatic individuals tested positive would be quarantined while symptomatic individuals tested positive would be sent to hospital for isolation

Scenario 5 - All participants are tested before attending the event, and asymptomatic individuals tested positive would be quarantined while symptomatic individuals tested positive would be sent to hospital for isolation

Scenario 6 - Same as scenario 5, and all participants are tested again on day 7 following opening of the event

**Pre-infectious local participants (preinfEvent)**

$\frac{dpreinfEvent}{\mathrm{dt}}= \lambda p*SusEvent\left[ t \right]- \left( \frac{1}{\mathrm{latent}} \right)* preinfEvent\left[ t \right]+ GDinf[t]* preinf[t] ;$- for scenarios 1, 2, 4-6

$\frac{dpreinfEvent}{\mathrm{dt}}= \lambda p*SusEvent\left[ t \right]- \left( \frac{1}{\mathrm{latent}} \right)* preinfEvent\left[ t \right];$- for scenario 3

**Asymptomatic local participants (AinfEvent)**

$\frac{dAinfEvent}{\mathrm{dt}}=asyp * \left( \frac{1}{\mathrm{latent}}* preinfEvent\left[ t \right] \right)- \frac{1}{r2}* AinfEvent\left[ t \right]+GDinf\left[ t \right]* Ainf\left[ t \right];$ -for scenarios 1 and 2

$$\frac{dAinfEvent}{\mathrm{dt}}=asyp * \left( \frac{1}{\mathrm{latent}}* preinfEvent\left[ t \right] \right)- \frac{1}{r2}* AinfEvent\left[ t \right];$$

-for scenario 3

$\frac{dAinfEvent}{\mathrm{dt}}=asyp * \left( \frac{1}{\mathrm{latent}}* preinfEvent\left[ t \right] \right)- \frac{1}{r2}* AinfEvent\left[ t \right]+GDinf\left[ t \right]* Ainf\left[ t \right]*(1-testcov);$ -for scenarios 4 and 5

$\frac{dAinfEvent}{\mathrm{dt}}=asyp * \left( \frac{1}{\mathrm{latent}}* preinfEvent\left[ t \right] \right)- \frac{1}{r2}* AinfEvent\left[ t \right]+GDinf\left[ t \right]* Ainf\left[ t \right]*\left( 1-testcov \right)-AinfEvent\left[ t=163 \right]*testcov;$ -for scenario 6

**Symptomatic local participants (infEvent)**

$\frac{dinfEvent}{\mathrm{dt}}=\left( 1-asyp \right)* \left( \frac{1}{\mathrm{latent}} * preinfEvent\left[ t \right] \right)-\frac{1}{dx+Infb4onset}* infEvent\left[ t \right]+ GDinf\left[ t \right]* inf[t];$ -for scenarios 1 and 2

$\frac{dinfEvent}{\mathrm{dt}}=\left( 1-asyp \right)* \left( \frac{1}{\mathrm{latent}} * preinfEvent\left[ t \right] \right)-\frac{1}{dx+Infb4onset}* infEvent\left[ t \right];$ -for scenario 3

$\frac{dinfEvent}{\mathrm{dt}}=\left( 1-asyp \right)* \left( \frac{1}{\mathrm{latent}} * preinfEvent\left[ t \right] \right)-\frac{1}{dx+Infb4onset}* infEvent\left[ t \right]+ GDinf\left[ t \right]* inf\left[ t \right]*(1-testcov);$ -for scenarios 4 and 5

$\frac{dinfEvent}{\mathrm{dt}}=\left( 1-asyp \right)* \left( \frac{1}{\mathrm{latent}} * preinfEvent\left[ t \right] \right)-\frac{1}{dx+Infb4onset}* infEvent\left[ t \right]+ GDinf\left[ t \right]* inf\left[ t \right]*\left( 1-testcov \right)-infEvent\left[ t=163 \right]*testcov;$ -for scenario 6

**Hospital local participants (HospEvent)**

$\frac{dHospEvent}{\mathrm{dt}}=\frac{1}{dx+Infb4onset}* \left( infEvent\left[ t \right] \right) -\left( cc+\frac{1}{r} \right)* HospEvent\left[ t \right];$ - for scenarios 1 and 2

$\frac{dHospEvent}{\mathrm{dt}}=\frac{1}{dx+Infb4onset}* \left( infEvent\left[ t \right] \right) -\left( cc+\frac{1}{r} \right)* HospEvent\left[ t \right]+ GDinf[t]* inf[t] ;$ - for scenario 3

$\frac{dHospEvent}{\mathrm{dt}}=\frac{1}{dx+Infb4onset}* \left( infEvent\left[ t \right] \right) -\left( cc+\frac{1}{r} \right)* HospEvent\left[ t \right]+ GDinf[t]* inf[t] *testcov;$ - for scenarios 4 and 5

$\frac{dHospEvent}{\mathrm{dt}}=\frac{1}{dx+Infb4onset}* \left( infEvent\left[ t \right] \right) -\left( cc+\frac{1}{r} \right)* HospEvent\left[ t \right]+ GDinf\left[ t \right]* inf\left[ t \right]*testcov+infEvent\left[ t=163 \right]*testcov;$ - for scenario 6

**ICU local participants (ICUEvent)**

$\frac{dICUEvent}{\mathrm{dt}}=cc * HospEvent\left[ t \right]- \left( \frac{1}{r}+ m \right)* ICUEvent\left[ t \right];$

**Recovery local participants (RecoveryEvent)**

$\frac{\mathrm{dRecoveryEvent}}{\mathrm{dt}}=\frac{1}{r}*\left( \mathrm{HospEvent}\left[ t \right]+ ICUEvent\left[ t \right] \right)+ \frac{1}{r2}*\left( \mathrm{AinfEvent}\left[ t \right] \right);$-for scenarios 1 and 2

$\frac{\mathrm{dRecoveryEvent}}{\mathrm{dt}}=\frac{1}{r}*\left( \mathrm{HospEvent}\left[ t \right]+ ICUEvent\left[ t \right] \right)+ \frac{1}{r2}*\left( \mathrm{AinfEvent}\left[ t \right] \right)+GDinf\left[ t-14 \right]* Ainf1\left[ t-14 \right]+asyp * GDinf[t-14]* preinf1[t-14];$-for scenario 3

$\frac{\mathrm{dRecoveryEvent}}{\mathrm{dt}}=\frac{1}{r}*\left( \mathrm{HospEvent}\left[ t \right]+ ICUEvent\left[ t \right] \right)+ \frac{1}{r2}*\left( \mathrm{AinfEvent}\left[ t \right] \right)+GDinf\left[ t-14 \right]* Ainf1\left[ t-14 \right]*testcov;$-for scenarios 4 and 5

$\frac{\mathrm{dRecoveryEvent}}{\mathrm{dt}}=\frac{1}{r}*\left( \mathrm{HospEvent}\left[ t \right]+ ICUEvent\left[ t \right] \right)+ \frac{1}{r2}*\left( \mathrm{AinfEvent}\left[ t \right] \right)+GDinf\left[ t-14 \right]* Ainf1\left[ t-14 \right]*testcov+AinfEvent\left[ if t=117, then t-14 \right]*testcov;$-for scenario 6

**Susceptible non-local participants (SusEventO)**

$\frac{d\mathrm{SusEventO}}{\mathrm{dt}}=(1-oPinf)*oPpar[t] -\lambda p*SusEventO\left[ t \right]+ overseaspar[t]* (1-seainf)$; - for scenario 1

$\frac{d\mathrm{SusEventO}}{\mathrm{dt}}=(1-oPinf)*oPpar[t] -\lambda p*SusEventO\left[ t \right]+overseaspar[t-14]* (1-seainf)$; - for scenarios 2 and 4

$\frac{d\mathrm{SusEventO}}{\mathrm{dt}}=-\lambda p*SusEventO\left[ t \right]+ (overseaspar\left[ t-14 \right]* \left( 1-seainf \right)+\left( 1-oPinf \right)*oPpar\left[ t-14 \right])$; - for scenario 3

$\frac{d\mathrm{SusEventO}}{\mathrm{dt}}=(1-oPinf)*oPpar[t] -\lambda p*SusEventO\left[ t \right]+ overseaspar[t]* (1-seainf)$; - for scenario 5

*oPinf* = prevalence of SARS-CoV-2 in participants and staff from other provinces; *oPpar*=number of participants and staff from other provinces; overseaspar = number of participants and staff from overseas; *seainf* = proportion of overseas participants tested positive

**Pre-infectious non-local participants (preinfEventO)**

$\frac{dpreinfEventO}{\mathrm{dt}}= \left( 1-Opos \right)*(oPinf* oPpar\left[ t \right]+overseaspar\left[ t \right]* seainf)+\lambda p*SusEventO\left[ t \right]- \left( \frac{1}{\mathrm{latent}} \right)* preinfEventO\left[ t \right];$ -for scenario 1

$\frac{dpreinfEventO}{\mathrm{dt}}= oPinf*(1-Opos) * oPpar\left[ t \right]+\lambda p*SusEventO\left[ t \right]- \left( \frac{1}{\mathrm{latent}} \right)* preinfEventO\left[ t \right];$ -for scenarios 2 and 4

$\frac{dpreinfEventO}{\mathrm{dt}}= \lambda p*SusEventO\left[ t \right]- \left( \frac{1}{\mathrm{latent}} \right)* preinfEventO\left[ t \right];$ -for scenario 3

$\frac{dpreinfEventO}{\mathrm{dt}}=(oPinf* oPpar\left[ t \right]+overseaspar[t]*seainf)*(1-Opos) +\lambda p*SusEventO\left[ t \right]- \left( \frac{1}{\mathrm{latent}} \right)* preinfEventO\left[ t \right];$ -for scenario 5

*Opos*=proportion of infected participants and staff from Mainland China who are tested positive

**Asymptomatic non-local participants (AinfEventO)**

$\frac{dAinfEventO}{\mathrm{dt}}=asyp * \left( \frac{1}{\mathrm{latent}}* preinfEventO\left[ t \right] \right)- \frac{1}{r2}* AinfEventO\left[ t \right]+asyp * Opos *(oPinf *oPpar[t]+ overseaspar[t]* seainf);$ -for scenario 1

$\frac{dAinfEventO}{\mathrm{dt}}=asyp * \left( \frac{1}{\mathrm{latent}}* preinfEventO\left[ t \right] \right)- \frac{1}{r2}* AinfEventO\left[ t \right]+asyp * oPinf * Opos * oPpar[t];$ -for scenario 2

$\frac{dAinfEventO}{\mathrm{dt}}=asyp * \left( \frac{1}{\mathrm{latent}}* preinfEventO\left[ t \right] \right)- \frac{1}{r2}* AinfEventO\left[ t \right];$ -for scenario 3

$\frac{dAinfEventO}{\mathrm{dt}}=asyp * \left( \frac{1}{\mathrm{latent}}* preinfEventO\left[ t \right] \right)- \frac{1}{r2}* AinfEventO\left[ t \right]+asyp * oPinf * Opos * oPpar\left[ t \right]*(1-testcov);$ -for scenario 4

$\frac{dAinfEventO}{\mathrm{dt}}=asyp * \left( \frac{1}{\mathrm{latent}}* preinfEventO\left[ t \right] \right)- \frac{1}{r2}* AinfEventO\left[ t \right]+asyp * Opos *\left( 1-testcov \right)*(oPinf *oPpar\left[ t \right]+ overseaspar[t]*seainf);$ -for scenario 5

**Symptomatic non-local participants (infEventO)**

$\frac{dinfEventO}{\mathrm{dt}}=\left( 1-asyp \right)*Opos*(oPinf*oPpar\left[ t \right]+ overseaspar[t]* seainf)+\left( 1-asyp \right)* \left( \frac{1}{\mathrm{latent}} * preinfEventO\left[ t \right] \right)-\frac{1}{dx+Infb4onset}* infEventO\left[ t \right];$ - for scenario 1

$\frac{dinfEventO}{\mathrm{dt}}=\left( 1-asyp \right)*oPinf*Opos* oPpar\left[ t \right]+\left( 1-asyp \right)* \left( \frac{1}{\mathrm{latent}} * preinfEventO\left[ t \right] \right)-\frac{1}{dx+Infb4onset}* infEventO\left[ t \right];$ - for scenario 2

$\frac{dinfEventO}{\mathrm{dt}}=\left( 1-asyp \right)* \left( \frac{1}{\mathrm{latent}} * preinfEventO\left[ t \right] \right)-\frac{1}{dx+Infb4onset}* infEventO\left[ t \right];$ - for scenario 3

$\frac{dinfEventO}{\mathrm{dt}}=\left( 1-asyp \right)*oPinf*Opos* oPpar\left[ t \right]*(1-testcov)+\left( 1-asyp \right)* \left( \frac{1}{\mathrm{latent}} * preinfEventO\left[ t \right] \right)-\frac{1}{dx+Infb4onset}* infEventO\left[ t \right];$ - for scenario 4

$\frac{dinfEventO}{\mathrm{dt}}=\left( 1-asyp \right)*Opos*(oPinf*oPpar\left[ t \right]+ overseaspar[t]*seainf)*(1-testcov)+\left( 1-asyp \right)* \left( \frac{1}{\mathrm{latent}} * preinfEventO\left[ t \right] \right)-\frac{1}{dx+Infb4onset}* infEventO\left[ t \right];$ - for scenario 5

**Quarantine participants before event (QGDEventb4)**

$\frac{dQGDEventb4}{\mathrm{dt}}=\mathrm{overseaspar}\left[ t \right]*( \left( 1-seainf \right)+seainf* (1-Opos))- overseaspar[t-14]*(\left( 1-seainf \right)+seainf* (1-Opos));$ - for scenario 2

$\frac{dQGDEventb4}{\mathrm{dt}}=\mathrm{overseaspar}\left[ t \right]*((1-seainf)+ seainf* (1-Opos))+(\left( 1-oPinf \right)+oPinf*(1-Opos))* oPpar\left[ t \right]+ GDinf\left[ t \right]* preinf\left[ t \right]+ GDinf\left[ t \right]* Ainf\left[ t \right]+GDpar[t]- overseaspar\left[ t-14 \right]* ((1-seainf)+ seainf* (1-Opos))-(\left( 1-oPinf \right)+oPinf*\left( 1-Opos \right))* oPpar\left[ t-14 \right]- GDinf\left[ t-14 \right]* preinf\left[ t-14 \right]- GDinf\left[ t-14 \right]* Ainf\left[ t-14 \right]+GDpar[t-14];$ - for scenario 3

$\frac{dQGDEventb4}{\mathrm{dt}}=\mathrm{overseaspar}\left[ t \right]* ((1-seainf)+ seainf* (1-Opos))+\mathrm{GDinf}\left[ t \right]* Ainf\left[ t \right]*testcov+asyp* Opos * oPinf * oPpar\left[ t \right]*testcov- overseaspar\left[ t-14 \right]* ((1-seainf)+ seainf* (1-Opos))- \mathrm{GDinf}\left[ t-14 \right]* Ainf\left[ t-14 \right]*testcov- asyp* Opos * oPinf * oPpar\left[ t-14 \right]*testcov;$ - for scenario 4

$\frac{dQGDEventb4}{\mathrm{dt}}=\mathrm{GDinf}\left[ t \right]* Ainf\left[ t \right]*testcov+asyp* Opos *(oPinf * oPpar\left[ t \right]+overseaspar\left[ t \right]*seainf )*testcov- \mathrm{GDinf}\left[ t-14 \right]* Ainf\left[ t-14 \right]*testcov- asyp* Opos *( oPinf * oPpar\left[ t-14 \right]+overseaspar\left[ t-14 \right]*seainf)*testcov;$ - for scenario 5

$\frac{dQGDEventb4}{\mathrm{dt}}=\mathrm{GDinf}\left[ t \right]* Ainf\left[ t \right]*testcov+AinfEvent\left[ t=163 \right]*testcov+asyp* Opos *\left( oPinf * oPpar\left[ t \right]+overseaspar\left[ t \right]*seainf \right)*testcov+AinfEventO\left[ t=163 \right]*testcov- \mathrm{GDinf}\left[ t-14 \right]* Ainf\left[ t-14 \right]*testcov-AinfEvent\left[ if t=177, then t=14 \right]*testcov- asyp* Opos *\left( oPinf * oPpar\left[ t-14 \right]+overseaspar\left[ t-14 \right]*seainf \right)*testcov-AinfEventO\left[ if t=177, then t-14 \right]*testcov;$ - for scenario 6

**Hospital non-local participants (HospEventO)**

$\frac{dHospEventO}{\mathrm{dt}}=\frac{1}{dx+Infb4onset}* \left( infEventO\left[ t \right] \right) -\left( cc+\frac{1}{r} \right)* HospEventO\left[ t \right];$ - for scenario 1

$\frac{dHospEventO}{\mathrm{dt}}=\frac{1}{dx+Infb4onset}* \left( infEventO\left[ t \right] \right) -\left( cc+\frac{1}{r} \right)* HospEventO\left[ t \right] +(1-asyp) *overseaspar\left[ t-14 \right]* seainf* (1-Opos)+overseaspar[t]*Opos* seainf ;$ - for scenario 2

$\frac{dHospEventO}{\mathrm{dt}}=\frac{1}{dx+Infb4onset}* \left( infEventO\left[ t \right] \right) -\left( cc+\frac{1}{r} \right)* HospEventO\left[ t \right]+\left( 1-asyp \right)* \left( \mathrm{overseaspar}\left[ t-14 \right]* seainf* \left( 1-Opos \right)+overseaspar\left[ t \right]* Opos *seainf+oPinf* \left( 1-Opos \right)* oPpar\left[ t-14 \right] \right) + oPinf*Opos* oPpar[t];$ - for scenario 3

$\frac{dHospEventO}{\mathrm{dt}}=\frac{1}{dx+Infb4onset}* \left( infEventO\left[ t \right] \right) -\left( cc+\frac{1}{r} \right)* HospEventO\left[ t \right]+\left( 1-asyp \right)* \left( \mathrm{overseaspar}\left[ t-14 \right]* seainf* (1-Opos) \right)+overseaspar\left[ t \right]* Opos *seainf +\left( 1-asy \right)*oPinf*Opos* oPpar\left[ t \right]*testcov;$ - for scenario 4

$\frac{dHospEventO}{\mathrm{dt}}=\frac{1}{dx+Infb4onset}* \left( infEventO\left[ t \right] \right) -\left( cc+\frac{1}{r} \right)* HospEventO\left[ t \right]+\left( 1-asy \right)*Opos*( oPinf*oPpar\left[ t \right]+ overseaspar[t]*seainf)*testcov;$ - for scenario 5

$\frac{dHospEventO}{\mathrm{dt}}=\frac{1}{dx+Infb4onset}* \left( infEventO\left[ t \right] \right) -\left( cc+\frac{1}{r} \right)* HospEventO\left[ t \right]+\left( 1-asy \right)*Opos*\left( oPinf*oPpar\left[ t \right]+ overseaspar\left[ t \right]*seainf \right)*testcov+infEventO\left[ t=163 \right]*testcov;$ - for scenario 6

**ICU non-local participants (ICUEventO)**

$\frac{dICUEventO}{\mathrm{dt}}=cc * HospEventO\left[ t \right]- \left( \frac{1}{r}+ m \right)* ICUEventO\left[ t \right];$

**Recovery non-local participants (RecoveryEventO)**

$\frac{\mathrm{dRecoveryEventO}}{\mathrm{dt}}=\frac{1}{r}*\left( \mathrm{HospEventO}\left[ t \right]+ ICUEventO\left[ t \right] \right)+ \frac{1}{r2}*\left( \mathrm{AinfEventO}\left[ t \right] \right);$-for scenario 1

$\frac{\mathrm{dRecoveryEventO}}{\mathrm{dt}}=\frac{1}{r}*\left( \mathrm{HospEventO}\left[ t \right]+ ICUEventO\left[ t \right] \right)+ \frac{1}{r2}*\left( \mathrm{AinfEventO}\left[ t \right] \right)+asyp* overseaspar\left[ t-14 \right]*seainf*(1-Opos) ;$-for scenario 2

$\frac{\mathrm{dRecoveryEventO}}{\mathrm{dt}}=\frac{1}{r}*\left( \mathrm{HospEventO}\left[ t \right]+ ICUEventO\left[ t \right] \right)+ \frac{1}{r2}*\left( \mathrm{AinfEventO}\left[ t \right] \right)+asyp*\left( 1-Opos \right)* (overseaspar\left[ t-14 \right]*seainf+oPinf* oPpar[t-14]);$-for scenario 3

$\frac{\mathrm{dRecoveryEventO}}{\mathrm{dt}}=\frac{1}{r}*\left( \mathrm{HospEventO}\left[ t \right]+ ICUEventO\left[ t \right] \right)+ \frac{1}{r2}*\left( \mathrm{AinfEventO}\left[ t \right] \right)+asyp* overseaspar\left[ t-14 \right]*seainf* \left( 1-Opos \right)+asyp* Opos * oPinf * oPpar\left[ t-14 \right]*testcov;$-for scenario 4

$\frac{\mathrm{dRecoveryEventO}}{\mathrm{dt}}=\frac{1}{r}*\left( \mathrm{HospEventO}\left[ t \right]+ ICUEventO\left[ t \right] \right)+ \frac{1}{r2}*\left( \mathrm{AinfEventO}\left[ t \right] \right)+asyp* Opos *(oPinf * oPpar\left[ t-14 \right]+overseaspar[t-14]*seainf)*testcov;$-for scenario 5

$\frac{\mathrm{dRecoveryEventO}}{\mathrm{dt}}=\frac{1}{r}*\left( \mathrm{HospEventO}\left[ t \right]+ ICUEventO\left[ t \right] \right)+ \frac{1}{r2}*\left( \mathrm{AinfEventO}\left[ t \right] \right)+asyp* Opos *\left( oPinf * oPpar\left[ t-14 \right]+overseaspar\left[ t-14 \right]*seainf \right)*testcov+AinfEventO\left[ if t=177, then t-14 \right]*testcov;$-for scenario 6

**Death (death)**

$\frac{\mathrm{dDeath}}{\mathrm{dt}}=m*( ICU\left[ t \right]+ ICUEvent\left[ t \right]+ICUEventO\left[ t \right]);$

**Community population (N)**

$$N\left[ t \right]= S\left[ t \right]+ preinf\left[ t \right]+ Ainf\left[ t \right]+ inf\left[ t \right]+ Hosp\left[ t \right]+ HospEvent\left[ t \right]+ HospEventO\left[ t \right]+ ICU\left[ t \right]+ ICUEvent\left[ t \right]+ ICUEventO\left[ t \right]+Recovery\left[ t \right] +QGD\left[ t \right]+QGDEventb4[t];$$

Community population include all population not actively participating in the event, including event participants sent to hospital, ICU or quarantine locations.

**Event population (EventN)**

$$eventN\left[ t \right]= SusEvent\left[ t \right]+ SusEventO\left[ t \right]+ preinfEvent\left[ t \right]+ preinfEventO\left[ t \right]+ AinfEvent\left[ t \right]+ AinfEventO\left[ t \right]+ infEvent\left[ t \right] + infEventO\left[ t \right]+ RecoveryEvent\left[ t \right]+ RecoveryEventO\left[ t \right];$$

Event participants sent to hospital, ICU or quarantine locations are no longer considered as active event population, but they are counted after recovery.

**Counter for number of newly confirmed cases (confirm)**

$\frac{\mathrm{dconfirm}}{\mathrm{dt}}=\frac{1}{dx+Infb4onset}* \left( \inf\left[ t \right]+infEvent\left[ t \right]+infEventO\left[ t \right] \right) + \left( 1-asyp \right)* Qdx *Qtesting\left[ t-14 \right]* \left( 1-posqtest\left[ t-14 \right] \right)+flowtracing\left[ t \right]*closepos*\mathrm{closepos}_{\mathrm{sym}}*\left( 1-closepreinf \right)+ Qtesting\left[ t \right]* posqtest\left[ t \right]+flowtracing\left[ t-3 \right]*closepos*\mathrm{closepos}_{\mathrm{sym}}*closepreinf;$ - for scenario 1

$\frac{\mathrm{dconfirm}}{\mathrm{dt}}=\frac{1}{dx+Infb4onset}* \left( \inf\left[ t \right]+infEvent\left[ t \right]+infEventO\left[ t \right] \right) + \left( 1-asyp \right)* Qdx *Qtesting\left[ t-14 \right]* \left( 1-posqtest\left[ t-14 \right] \right)+flowtracing\left[ t \right]*closepos*\mathrm{closepos}_{\mathrm{sym}}*\left( 1-closepreinf \right)+ Qtesting\left[ t \right]* posqtest\left[ t \right]+flowtracing\left[ t-3 \right]*closepos*\mathrm{closepos}_{\mathrm{sym}}*closepreinf +(1-asyp) *overseaspar\left[ t-14 \right]* seainf* (1-Opos)+overseaspar[t]*Opos* seainf ;$- for scenario 2

$\frac{\mathrm{dconfirm}}{\mathrm{dt}}=\frac{1}{dx+Infb4onset}* \left( \inf\left[ t \right]+infEvent\left[ t \right]+infEventO\left[ t \right] \right) + \left( 1-asyp \right)* Qdx *Qtesting\left[ t-14 \right]* \left( 1-posqtest\left[ t-14 \right] \right)+flowtracing\left[ t \right]*closepos*\mathrm{closepos}_{\mathrm{sym}}*\left( 1-closepreinf \right)+ Qtesting\left[ t \right]* posqtest\left[ t \right]+flowtracing\left[ t-3 \right]*closepos*\mathrm{closepos}_{\mathrm{sym}}*closepreinf +\left( 1-asyp \right)* \left( \mathrm{overseaspar}\left[ t-14 \right]* seainf* \left( 1-Opos \right)+oPinf* \left( 1-Opos \right)* oPpar\left[ t-14 \right] \right)+overseaspar\left[ t \right]* Opos *seainf + oPinf*Opos* oPpar\left[ t \right]+ GDinf\left[ t \right]* inf\left[ t \right];$ -for scenario 3

$\frac{\mathrm{dconfirm}}{\mathrm{dt}}=\frac{1}{dx+Infb4onset}* \left( \inf\left[ t \right]+infEvent\left[ t \right]+infEventO\left[ t \right] \right) + \left( 1-asyp \right)* Qdx *Qtesting\left[ t-14 \right]* \left( 1-posqtest\left[ t-14 \right] \right)+flowtracing\left[ t \right]*closepos*\mathrm{closepos}_{\mathrm{sym}}*\left( 1-closepreinf \right)+ Qtesting\left[ t \right]* posqtest\left[ t \right]+flowtracing\left[ t-3 \right]*closepos*\mathrm{closepos}_{\mathrm{sym}}*closepreinf +\left( 1-asyp \right)* \left( \mathrm{overseaspar}\left[ t-14 \right]* seainf* (1-Opos) \right)+overseaspar\left[ t \right]* Opos *seainf +\left( 1-asy \right)*oPinf*Opos* oPpar\left[ t \right]*testcov+ GDinf[t]* inf[t] *testcov;$ -for scenario 4

$\frac{\mathrm{dconfirm}}{\mathrm{dt}}=\frac{1}{dx+Infb4onset}* \left( \inf\left[ t \right]+infEvent\left[ t \right]+infEventO\left[ t \right] \right) + \left( 1-asyp \right)* Qdx *Qtesting\left[ t-14 \right]* \left( 1-posqtest\left[ t-14 \right] \right)+flowtracing\left[ t \right]*closepos*\mathrm{closepos}_{\mathrm{sym}}*\left( 1-closepreinf \right)+ Qtesting\left[ t \right]* posqtest\left[ t \right]+flowtracing\left[ t-3 \right]*closepos*\mathrm{closepos}_{\mathrm{sym}}*closepreinf +\left( 1-asy \right)*Opos*( oPinf*oPpar\left[ t \right]+ overseaspar[t]*seainf)*testcov+ GDinf[t]* inf[t] *testcov;$ -for scenario 5

$\frac{\mathrm{dconfirm}}{\mathrm{dt}}=\frac{1}{dx+Infb4onset}* \left( \inf\left[ t \right]+infEvent\left[ t \right]+infEventO\left[ t \right] \right) + \left( 1-asyp \right)* Qdx *Qtesting\left[ t-14 \right]* \left( 1-posqtest\left[ t-14 \right] \right)+flowtracing\left[ t \right]*closepos*\mathrm{closepos}_{\mathrm{sym}}*\left( 1-closepreinf \right)+ Qtesting\left[ t \right]* posqtest\left[ t \right]+flowtracing\left[ t-3 \right]*closepos*\mathrm{closepos}_{\mathrm{sym}}*closepreinf +\left( 1-asy \right)*Opos*\left( oPinf*oPpar\left[ t \right]+ overseaspar\left[ t \right]*seainf \right)*testcov+ GDinf\left[ t \right]* inf\left[ t \right]*testcov+\left( \mathrm{infEvent}\left[ t=163 \right]+infEventO\left[ t=163 \right] \right)*testcov;$ -for scenario 6

**Counter for number of newly infected cases (GDinf)**

$$\frac{\mathrm{dGD}inf}{\mathrm{dt}}=\lambda*(S\left[ t \right]+SusEvent\left[ t \right]+SusEventO\left[ t \right]);$$

# **Maximum likelihood estimation**

Coefficients:

Estimate Std. Error

asyrate 0.33899 0.0179

controlr2 0.90 0.0464

-2 log L: 504.6972

Where *asyrate* refers to the proportion of asymptomatic infections; *controlr2* refers to contact rate reduction comparing with pre-epidemic level from 9 Feb to 23 Feb 2020

# **Sensitivity analysis**

To account for variability of R0, contact rate, and proportion of asymptomatic infections over time, we have performed 5000 simulations with random selection of R0 between 1 and 3, contact rate reduction between 0% and 99% comparing with pre-epidemic level, and proportion of asymptomatic infections between 20% and 80%. The simulated values were used to calculate interquartile range (IQR) of total new infections and cumulative number of reported cases.

# **eTable 2 Sensitivity analysis: contacts tripled and decupled in the event comparing with the community**

| **Contact rate in the event** |  | **S1** | **S2** | **S3** | **S4** | **S5** | **S6** |
| --- | --- | --- | --- | --- | --- | --- | --- |
| **Double** | cumulative number new infections^ | 1243 | 744 | 644 | 648 | 663 | 656 |
|  | % ^#^ | 93% | 16% | 0% | 1% | 3% | 2% |
| **Triple** | cumulative number total new infections^ | 2159 | 893 | 644 | 651 | 685 | 670 |
|  | % ^#^ | 235% | 39% | 0% | 1% | 6% | 4% |
| **Ten-fold** | cumulative number total new infections^ | 86544 | 16493 | 644 | 894 | 2126 | 1640 |
|  | % ^#^ | 13339% | 2461% | 0% | 39% | 230% | 155% |

^cumulative number new infections by 31 July 2020

# percentage change of cumulative number new infections comparing with basecase scenario by 31 July 2020

# **eFigure 1 Sensitivity analysis: impact of contact rate**

Percentage change of cumulative number of new infections and cumulative number of confirmed cases above basecase scenario by 31 July 2020, along the change of contact rate (10%-100% comparing with pre-epidemic level) from 1 May 2020


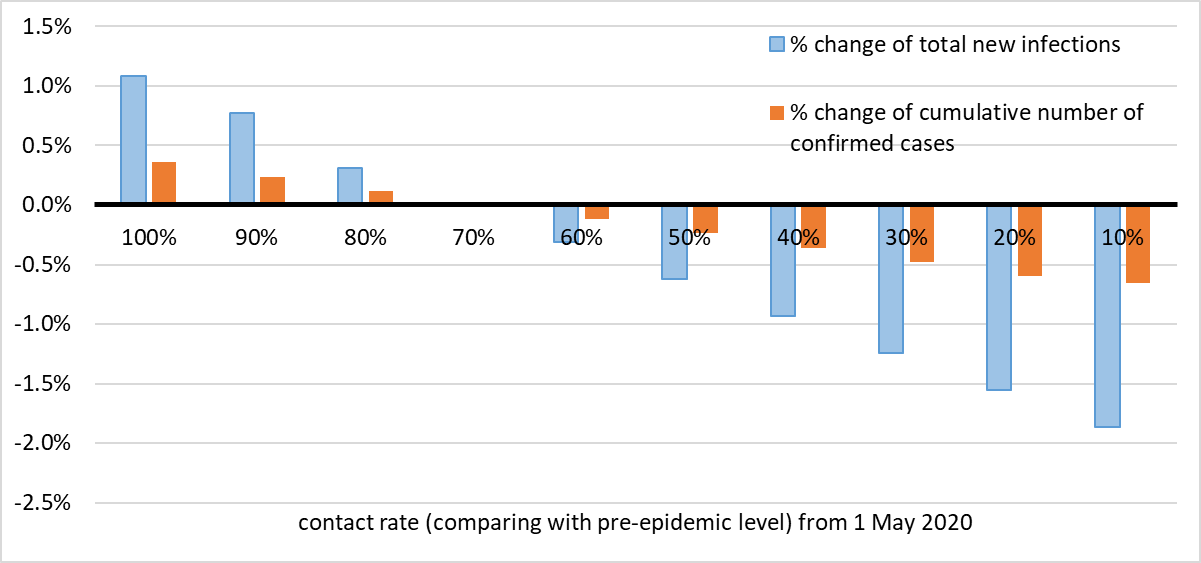


# **eFigure 2 Two-way sensitivity analysis: date of changing to higher contact rate and level of contact rate**

Percentage change of cumulative number of new infections above basecase scenario by 31 July 2020, along different date (x-axis) of changing from 10% contact rate from 9 February 2020 to higher contact rate (shown in color scale). Parameter values used in basecase scenario (24 February 2020, 70% contact rate) is shown in a red triangle


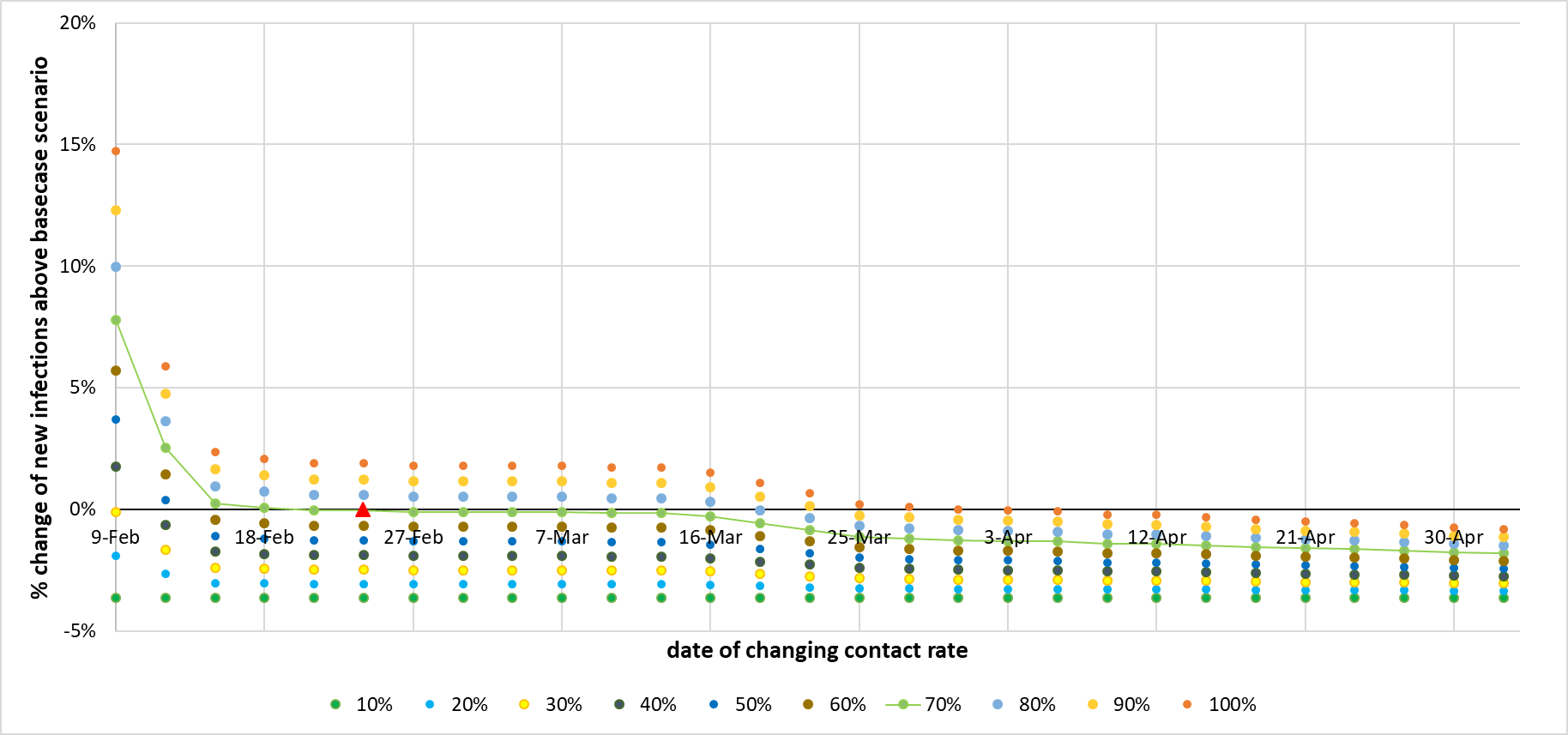


# **eFigure 3 Sensitivity analysis: impact of testing coverage in scenarios 4-6**

Percentage change of cumulative number of new infections above basecase scenario by 31 July 2020, along different testing coverage in participants in scenarios 4-6


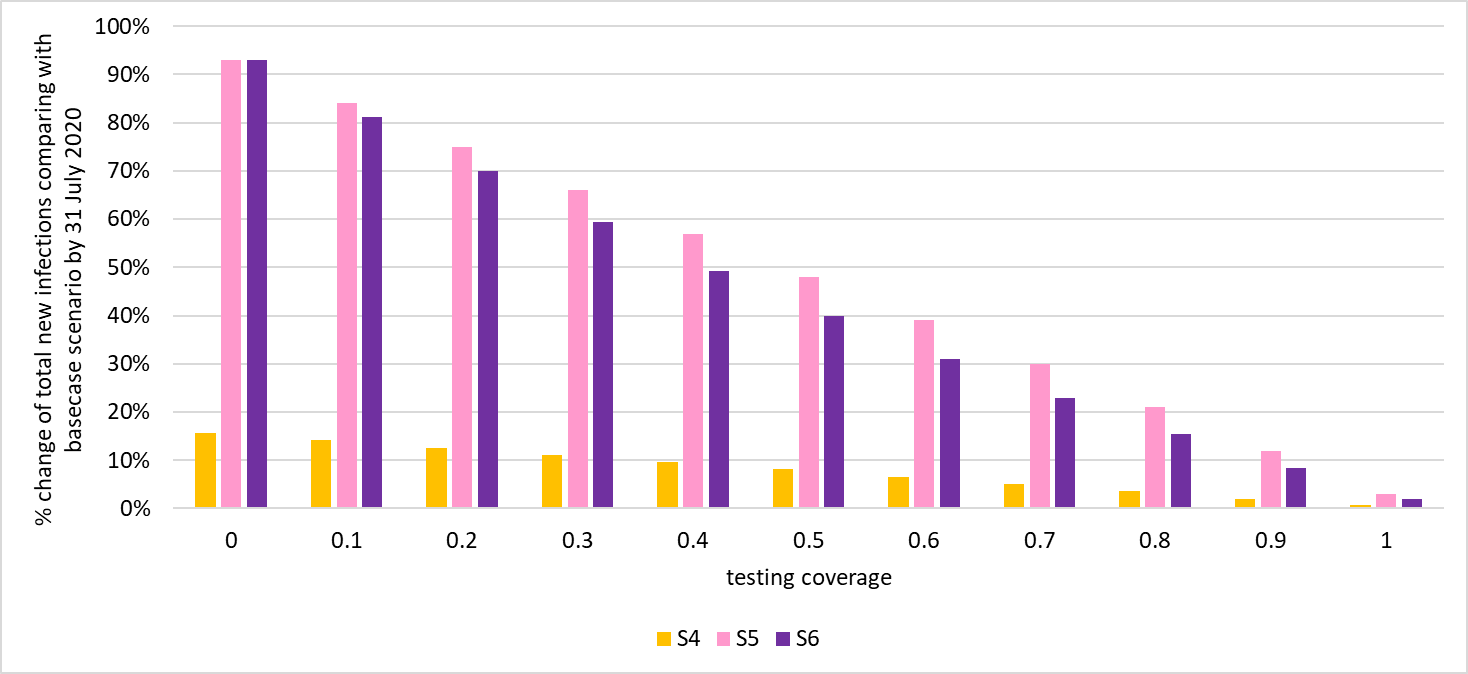


# **eFigure 4 Sensitivity analysis: impact of SARS-CoV-2 prevalence in participants before the event**

Percentage change of cumulative number of new infections above basecase scenario by 31 July 2020, along different prevalence in participants before event or proportion of infected cases participating in the event


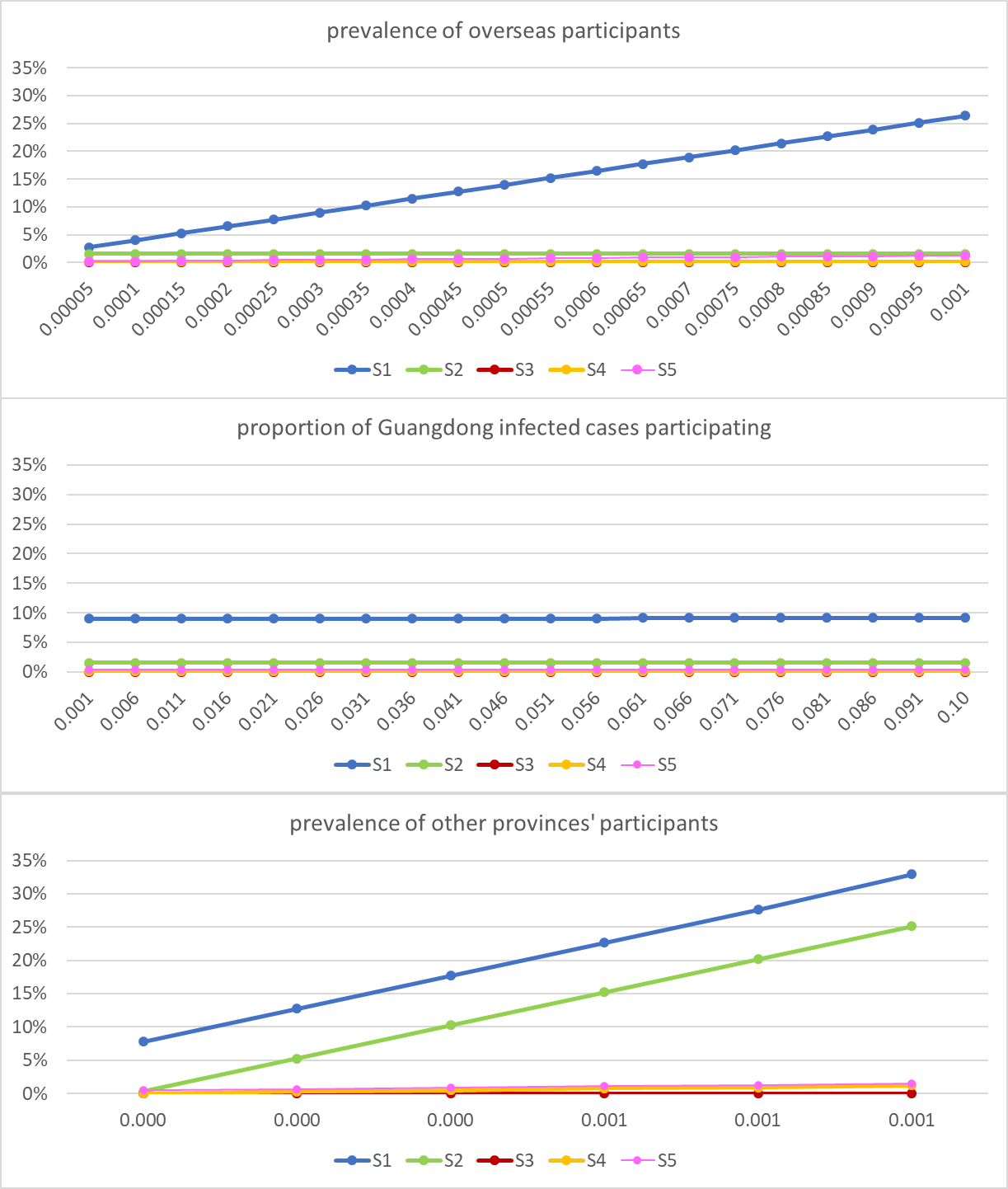


# **Reference**

[1] Health Commission of Guangdong Province. [Guangdong Province decides to activate the first level emergency response]. 2020. <http://wsjkw.gd.gov.cn/zwyw_yqxx/content/post_2878895.html> (accessed 30 May 2020)

[2] Li Q, Guan X, Wu P, et al. Early Transmission Dynamics in Wuhan, China, of Novel Coronavirus–Infected Pneumonia. *N Engl J Med*. 2020;382:1199-1207.

[3] Ferguson NM, Laydon D, Nedjati-Gilani G, et al. Impact of non-pharmaceutical interventions (NPIs) to reduce COVID-19 mortality and healthcare demand. Imperial College London (16-03-2020). <https://doi.org/10.25561/77482>

[4] Bi Q, Wu Y, Mei S, et al. Epidemiology and transmission of COVID-19 in 391 cases and 1286 of their close contacts in Shenzhen, China: a retrospective cohort study. *Lancet Infect Dis*. 2020; 20:911-919.
